# Chronic Infectious Complications of Recreational Urethral Sounding With Retained Foreign Body

**DOI:** 10.7759/cureus.9750

**Published:** 2020-08-14

**Authors:** Dubert M Guerrero, Aishwarya Sharma

**Affiliations:** 1 Infectious Diseases, Sanford Health, Fargo, USA; 2 Internal Medicine, University of North Dakota, Fargo, USA; 3 Infectious Disease, Sanford Health, Fargo, USA

**Keywords:** sounding, infection, diskitis

## Abstract

The practice of recreational urethral sounding involves insertion of foreign body in the urethra usually for sexual gratification. We present a case of a 62-year-old male with longstanding recurrent urinary tract infections complicated with Staphylococcus epidermidis bacteremia, discitis, and osteomyelitis at T12-L1 vertebral level associated with left psoas abscess secondary to a retained foreign body inserted into his urethra and urinary bladder. He had extraction of foreign body, cystoscopy, and open cystolithotripsy. He received long-term antibiotics and back surgery resulting in residual chronic back pain. This case illustrates important chronic infectious complications associated with the high-risk sexual practice of urethral sounding.

## Introduction

From a medical perspective, a sound is an instrument inserted into bodily passages most commonly the urethra or uterus to gently probe, dilate, or relieve strictures [1-2]. Beyond healthcare practice, urethral sounding or urethral "play" refers to the insertion of a foreign body into the urethra often for sexual or erotic purposes [3-5]. Occasionally, these foreign objects may end up within the urinary bladder. These could predispose to infection, injury, or trauma and require prompt treatment. Patients commonly present with acute symptoms including obstruction, hematuria, urinary frequency, dysuria, or pelvic pain [6]. Here, we report the consequences of a chronic intravesical foreign body that led to persistent bacteremia, psoas abscess, and deep spinal infectious complications in a 62-year-old man.

## Case presentation

**Figure 1:**
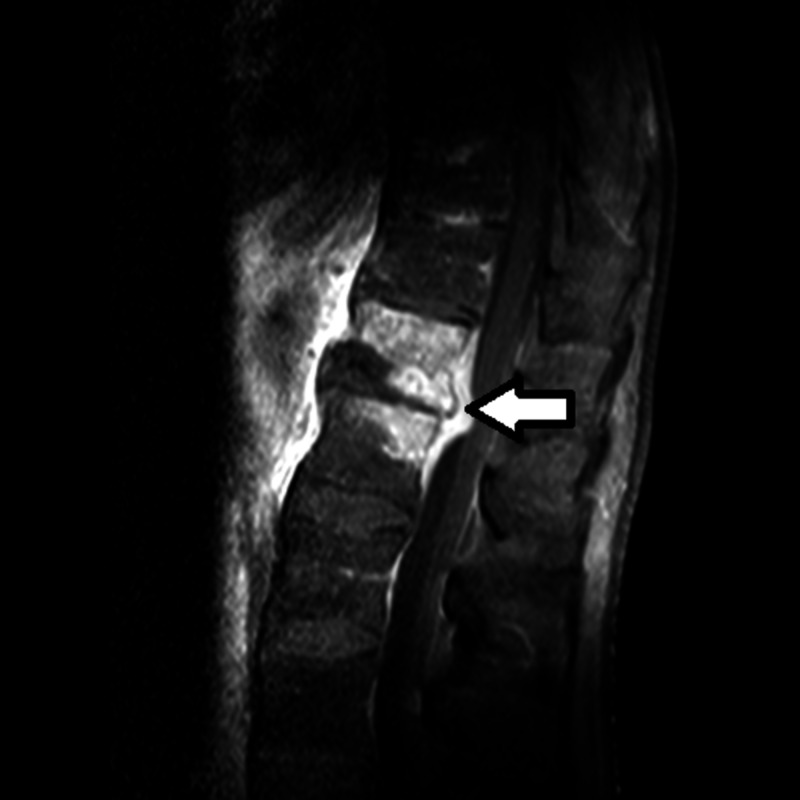
MRI of thoracolumbar area. Image shows abnormal enhancement at level T12-L1 consistent with discitis and osteomyelitis.

A 62-year-old male was admitted for evaluation and management of three weeks of progressive back pain radiating to his legs. He was noted to have a history of nicotine and amphetamine abuse, hypertension, mood disorder, and neuropathic pain. He denied any lower extremity weakness, bowel or urinary incontinence. Physical examination including neurologic assessment was unremarkable. White blood cell count was normal at 8.6 K/uL (reference range, 4-11) but inflammatory markers, erythrocyte sedimentation rate, and C-reactive protein were significantly elevated at 94 mm/h (reference range, 0-15) and 110 mg/L (reference range, 0-8) respectively. MRI of the back revealed abnormal marrow signal and enhancement in the T12-L1 vertebral bodies centered at the T12-L1 disc space likely secondary to discitis (Figure 1). There was corresponding abnormal paraspinal edema and enhancement with a probable 1.7 cm intramuscular abscess in the left psoas muscle. The abscess was aspirated and grew Staphylococcus epidermidis. The same organism was in multiple blood cultures. He was placed on IV cefazolin..

**Figure 2:**
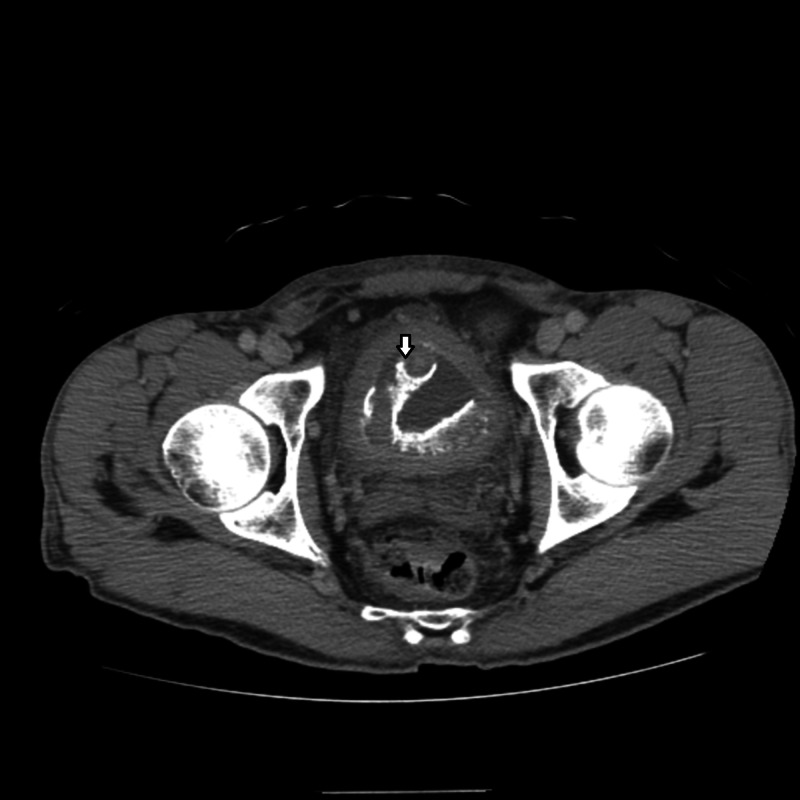
CT urogram demonstrating coiled tube like structure in the urinary bladder.

**Figure 3:**
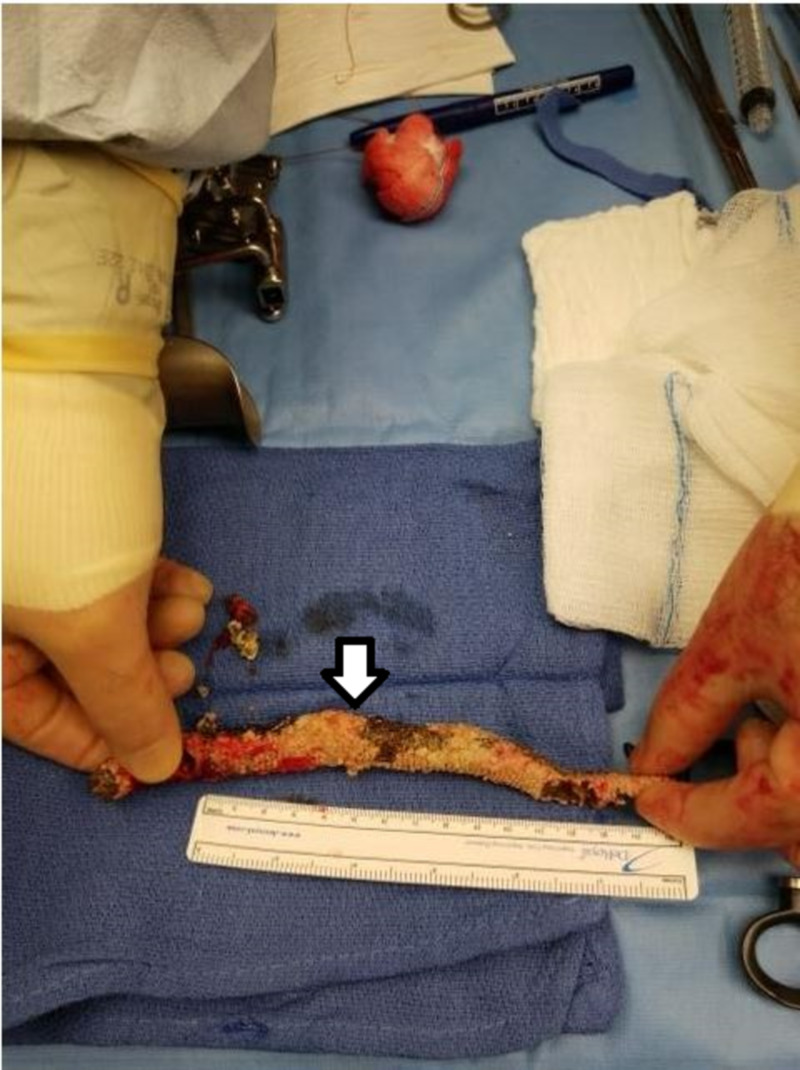
Foreign body extracted from open cystotomy. Calcified foreign body in the bladder is 16.9 cm in length and 1.2 cm in diameter.

He also complained of dysuria and hematuria. Further history revealed frequent urinary tract infections over the last six months with S. epidermidis as well for which he received different courses of oral antibiotics without relief. A CT urogram was done for persistent urinary symptoms and it identified a tubular 1.5 cm diameter peripherally calcified 10-12 cm structure with tapered distal ends and intermediate internal attenuation coiled in the urinary bladder (Figure 2). After careful history, he admitted his girlfriend inserted a sex toy shaped like a fishing worm into his urethra few months back, but he did not remember if it was removed. He underwent cystoscopy and open cystolithotripsy by which the foreign body was extracted (Figure 3). 

The patient was discharged to a skilled nursing facility to complete IV antibiotics for six weeks followed by oral cephalexin for additional six weeks. Repeat MRI showed destruction in the intervertebral disc between T12-L1 and paraspinal soft tissue enhancement. He eventually required a T12-L1 corpectomy and posterior instrumented fusion of T9-L3. The patient continued to have significant back pain even after a year of follow-up.

## Discussion

One of the most common reasons associated with the insertion of foreign bodies into the urethra is sexual or erotic in nature [5]. From a sexual context, this is referred to as urethral sounding. Our patient recalled his girlfriend inserting a sex toy into his urethra during sexual intercourse and it ended up in the urinary bladder. He did not seek consult for the retained device. However, he was repeatedly treated for urinary tract infection after that for months. It was only when he presented with sepsis that it was identified in imaging and retrieved. Unfortunately, the prolonged bacteremia resulted in discitis, osteomyelitis, psoas abscess, and prolonged debilitation from back pain.

Like our patient, most are hesitant to admit foreign body insertion and may present to a healthcare provider only when symptoms develop or if there are complications [7]. Acutely retained foreign body in the urinary bladder can cause irritation and patients may complain of frequency, dysuria, or pelvic pain. Hematuria is not uncommon especially if there is injury to urethral mucosal membrane or injury to the wall of the bladder. Obstruction and inability to void may occur especially if the urethra is involved [6-9]. Subacute and long-term intravesical or urethral foreign body may then eventually lead to frequent urinary tract infections, abscesses, calculus formation, urethral diverticuli, or stricture, or fistula formation [5-6, 10-11].

Diagnosis of retained foreign body in the bladder is challenging especially if patients do not volunteer the history that a foreign body was introduced. Patients may refuse answering questions on sexual history or avoid a genital examination. Radiologic imaging such as plain X-ray, ultrasound, or CT scan is warranted especially if the index of suspicion is high. It provides information on the extent, size, and location of the foreign body [5].

For definitive management, endoscopic and minimally invasive techniques should be encouraged. Most intravesical foreign bodies may be removed by this approach. Larger objects, calcified or those irretrievable by endoscopic approach may require open surgery such as cystotomy [7].

Several previous cases of intravesical foreign objects reported in literature usually presented with acute to subacute symptoms of bladder pains, irritation, urinary retention, or dysuria [12-16]. Our patient was also treated for recurrent urinary tract infections for over six months before he eventually ended up with bacteremia and deep-seated infectious complications of discitis and psoas abscess that was managed both medically and surgically. This case is unique as it highlights the potential for chronic infections and complications associated with a long-standing intravesical foreign body. Deep infections such as discitis and psoas abscess suggest prolonged period of bacteremia. The calcifications in the tubular structure found in the bladder also point towards its chronicity. Unfortunately, our patient ended up with chronic back pain as a sequela of foreign body retention and subsequent infection.

## Conclusions

Retained foreign body in the urinary bladder as a consequence of urethral sounding may be a rare occurrence. A number of these patients may not necessarily present acutely to the urology office or the emergency room but rather to primary healthcare providers complaining of recurrent urinary tract infections. A high index of suspicion is warranted to facilitate diagnosis. Long-term complications may include bacteremia resulting in infectious complications such as psoas abscess and discitis.
